# Endothelial NCK2 promotes atherosclerosis progression in male but not female *Nck1*-null atheroprone mice

**DOI:** 10.3389/fcvm.2022.955027

**Published:** 2022-08-12

**Authors:** Briana C. Bywaters, Gladys Pedraza, Andreea Trache, Gonzalo M. Rivera

**Affiliations:** ^1^Department of Veterinary Pathobiology, Texas A&M University, College Station, TX, United States; ^2^Department of Medical Physiology, Texas A&M Health Science Center, Bryan, TX, United States

**Keywords:** *Nck*, endothelium, sexual dimorphism, atherosclerosis, plaque burden, plaque inflammation, APOE-deficiency, high fat diet

## Abstract

A better understanding of endothelial dysfunction holds promise for more effective interventions for atherosclerosis prevention and treatment. Endothelial signaling by the non-catalytic region of the tyrosine kinase (NCK) family of adaptors, consisting of NCK1 and NCK2, has been implicated in cardiovascular development and postnatal angiogenesis but its role in vascular disease remains incompletely understood. Here, we report stage- and sex-dependent effects of endothelial NCK2 signaling on arterial wall inflammation and atherosclerosis development. Male and female *Nck1*-null atheroprone mice enabling inducible, endothelial-specific *Nck2* inactivation were fed a high fat diet (HFD) for 8 or 16 weeks to model atherosclerosis initiation and progression, respectively. Analysis of aorta preparations *en face* during disease progression, but not initiation, showed a significant reduction in plaque burden in males, but not females, lacking endothelial NCK2 relative to controls. Markers of vascular inflammation were reduced by endothelial NCK2 deficiency in both males and females during atherosclerosis progression but not initiation. At advanced stages of disease, plaque size and severity of atherosclerotic lesions were reduced by abrogation of endothelial NCK2 signaling only in males. Collectively, our results demonstrate stage- and sex-dependent modulation of atherosclerosis development by endothelial NCK2 signaling.

## Introduction

Endothelial dysfunction is a critical initiating event in the pathogenesis of atherosclerosis. It is well-established that atherosclerosis risk factors, in particular altered metabolism (1), promote a proinflammatory endothelium in regions of the arterial tree exposed to disturbed hemodynamics (2). It is also known that loss of atheroprotective endothelial functions is linked to increased expression of cell adhesion and chemoattractant molecules that foster the recruitment of leukocytes and their migration into the subintimal space (3). However, the mechanisms linking these risk factors to atherogenesis remain incompletely understood. Current therapies for atherosclerosis, relying primarily on reducing circulating levels of cholesterol (4) and inflammation (5), do not preserve or restore endothelial homeostasis. Such limitations, together with a shifting focus from treatment to prevention of atherosclerotic cardiovascular disease, underscore the need for a deeper understanding of the molecular mechanisms underpinning endothelial dysfunction.

The non-catalytic region of the tyrosine kinase (NCK) family of adaptors, consisting of NCK1 and NCK2, links tyrosine phosphorylation with cytoskeletal remodeling [reviewed in (6)]. Their architecture consists of three N-terminal Src Homology (SH)3 domains and a C-terminal SH2 domain tethered by unstructured linkers. NCK adaptors assemble multimolecular signaling complexes to promote endothelial cell migration (7) and morphogenesis (8) *in vitro*. Endothelial NCK signaling is required for cardiovascular development (9), postnatal retinal angiogenesis and pathological ocular neovascularization (10). Furthermore, NCK1 and NCK2 were shown to mediate oxidative stress-induced endothelial inflammation (11). Functional redundancy of NCK1 and NCK2 has been inferred by their conserved (~68% identity) amino acid sequence (12) and absence of an overt phenotype in mice lacking either one of the paralogues (13). However, recent studies suggest that NCK1, but not NCK2, mediates disturbed flow-induced endothelial permeability (14), as well as atherogenic inflammation and plaque formation in male mice (14). To our knowledge, the role of endothelial NCK signaling in disease stage- and sex-dependent regulation of arterial wall inflammation and atherogenesis has not been investigated. In the present study, we show that inducible, endothelial-specific deletion of NCK2 does not impact HFD-induced atherosclerosis initiation. During atherosclerosis progression, however, abrogation of endothelial NCK2 signaling ameliorates inflammation of the arterial wall in both sexes, whereas severity of lesions and atherosclerosis progression are reduced in male, but not female, *Nck1*-null atheroprone mice.

## Materials and methods

Information about antibodies and reagents is detailed in Supplementary materials and methods.

### Mice

Mice were maintained in the Comparative Medicine Program Laboratory Animal Resources and Research facility at Texas A&M University. Animal handling and experimental procedures were performed according to a protocol (AUP 2019-0184) approved by the Institutional Animal Care and Use Committee of Texas A&M University. *Nck*1^−/−^; *Nck*2^fl/fl^; *Cdh5*-Cre^ERT2^ mice were a kind gift from Anne Eichmann. These animals were generated by breeding *Nck*1^−/−^; *Nck*2^fl/fl^ mice (15, 16) with the *Cdh5*-Cre^ERT2^ line (17). We crossed *Nck*1^−/−^; *Nck*2^fl/fl^; *Cdh5*-Cre^ERT2^ mice with *ApoE*^−/−^ mice, purchased from The Jackson Laboratory (Bar Harbor, Maine), to generate mixed background *Nck*1^−/−^; *Nck*2^fl/fl^; ApoE^−/−^ mice with (eNck2-) and without (eNck2+) the *Cdh5*-Cre^ERT2^ driver. The Cre allele was passed through male breeders to prevent recombination in oocytes. DNA from tail snips was used for genotyping. At 8 weeks of age, all mice received a total of five intraperitoneal injections, administered every other day, of 75 mg/kg tamoxifen (Sigma-Aldrich) in 100 μL corn oil. After a 1-week recovery period, all mice were subjected to a HFD, containing 0.2% cholesterol and 21% total fat (Envigo TD.88137), and water *ad libitum*. Mice were maintained on a 12-h light/dark cycle. Both male and female animals were used in our study.

### Endothelial cell isolation and western blotting

Successful *Nck2* recombination was confirmed by detection of NCK protein levels in extracts from lung endothelial cells isolated as previously described (10). Briefly, lung tissue from two mice per genotype (eNck2+ and eNck2-) was enzymatically digested and endothelial cells were enriched with anti-CD31 coated Dynabeads. A second enrichment with anti-CD102-coated Dynabeads was performed before culturing cells at 37C° on fibronectin-coated tissue culture dishes in EGM-2 media (Lonza Walkersville). Cells (passage two) were lysed in kinase lysis buffer and mixed with 5X SDS loading buffer. The mixtures were boiled at 95C for 5 min. Proteins were separated by 12% SDS-PAGE and transferred to a nitrocellulose membrane for western blotting using antibodies against CD144, NCK, and β-actin.

### Blood serum assays

Mice were fasted for 16 h before blood sample collection *via* cardiac puncture into untreated tubes immediately following CO2 asphyxiation. Commercially available kits were used to determine serum levels of HDL fraction and total cholesterol (Invitrogen) and triglycerides (Invitrogen) following manufacturer's instructions.

### Tissue processing

To investigate atherosclerotic lesion properties, mice were perfused with ice cold DPBS followed by 10% non-buffered formalin (NBF) through the left ventricle. The heart, innominate artery, and thoracic aorta were collected and kept in 10% NBF at 4C° for 24 h. We chose to focus our analysis on the aortic root to detect incipient atherosclerotic lesions because lesion development tends to occur earlier at this site compared to other sites of the arterial tree (18). The innominate artery was the submerged in a 30% sucrose solution overnight at 4C before being mounted in OCT. Mounted tissues were cryosectioned into 5 um sections. Sections were stained with Picrosirius Red. The base of the heart was separated, and paraffin embedded 5 μm serial sections of the aortic root were collected for comparative analysis, as described previously (18). Slides were stained with H&E, Picrosirius Red, or used for fluorescence staining. Slides subjected to histologic staining were imaged in brightfield using an Olympus VS120 Virtual Slide Scanning System equipped with a U Plan S-Apo dry 10X objective. Fluorescence staining was performed using Isolectin-B4 and antibodies against ICAM-1, CD31, VCAM-1, Mac-2, and αSMA. Preparations subjected to fluorescence staining were imaged using an Olympus Fluoview FV3000 Confocal Laser Scanning Microscope equipped with a U Plan S-Apo dry10X objective.

### Atherosclerotic lesion assessment

Aortas were cleaned of adventitial fat and stained with Oil Red O (Fisher). Vessels were then opened *en face* and imaged (3X) using a 16-megapixel LG camera. Analysis of *en face* images from aortas was performed using ImageJ software. Analysis of aortic root sections was completed using QuPath software. Samples that did not display all three leaflets of the aortic valve across three serial slides were excluded from analysis. Lesion area was determined by subtracting the area of the lumen from the area delimited by the internal elastic lamina. The sum of lesion area across serial sections with visible valve leaflets was calculated. All other quantifications were performed using a single representative slide containing four serial sections for each sample. For quantification of collagen, Picrosirius Red signal was detected using a standardized pixel classification thresholder. Medial area was calculated as the difference between the areas delimited by the internal and external elastic laminae. Lesions were classified according to established criteria (19). For fluorescence images, a pixel classifier thresholder was developed for each marker and applied to all corresponding samples. For samples stained for CD31 and ICAM-1 as well as VCAM-1 and Mac-2, the neointima was traced by hand using the internal elastic lamina as the outer bound.

### Statistical analyses

Normal distribution assumptions were assessed using the Shapiro Test. Datasets that followed a normal distribution were subjected to two-way ANOVA followed by Tukey's multiple means comparison test. Datasets that did not follow a normal distribution were analyzed using Wilcoxon Rank Sum Test with Bonferroni correction. Differences were considered significant at *p* < 0.05. Statistical analysis and visualization were performed using R v4.0.5. Quantitative results are displayed using box plots, where the bottom and top horizontal lines in boxes indicate the 25^th^ and 75^th^ percentile, respectively, and the central line is the median. Whiskers extend 1.5 x the interquartile range and dots represent individual observations. Numbers of animals/group (n) are indicated in parentheses.

## Results

### Endothelial NCK2 accelerates atherosclerosis progression in male mice

**Figure 1:**
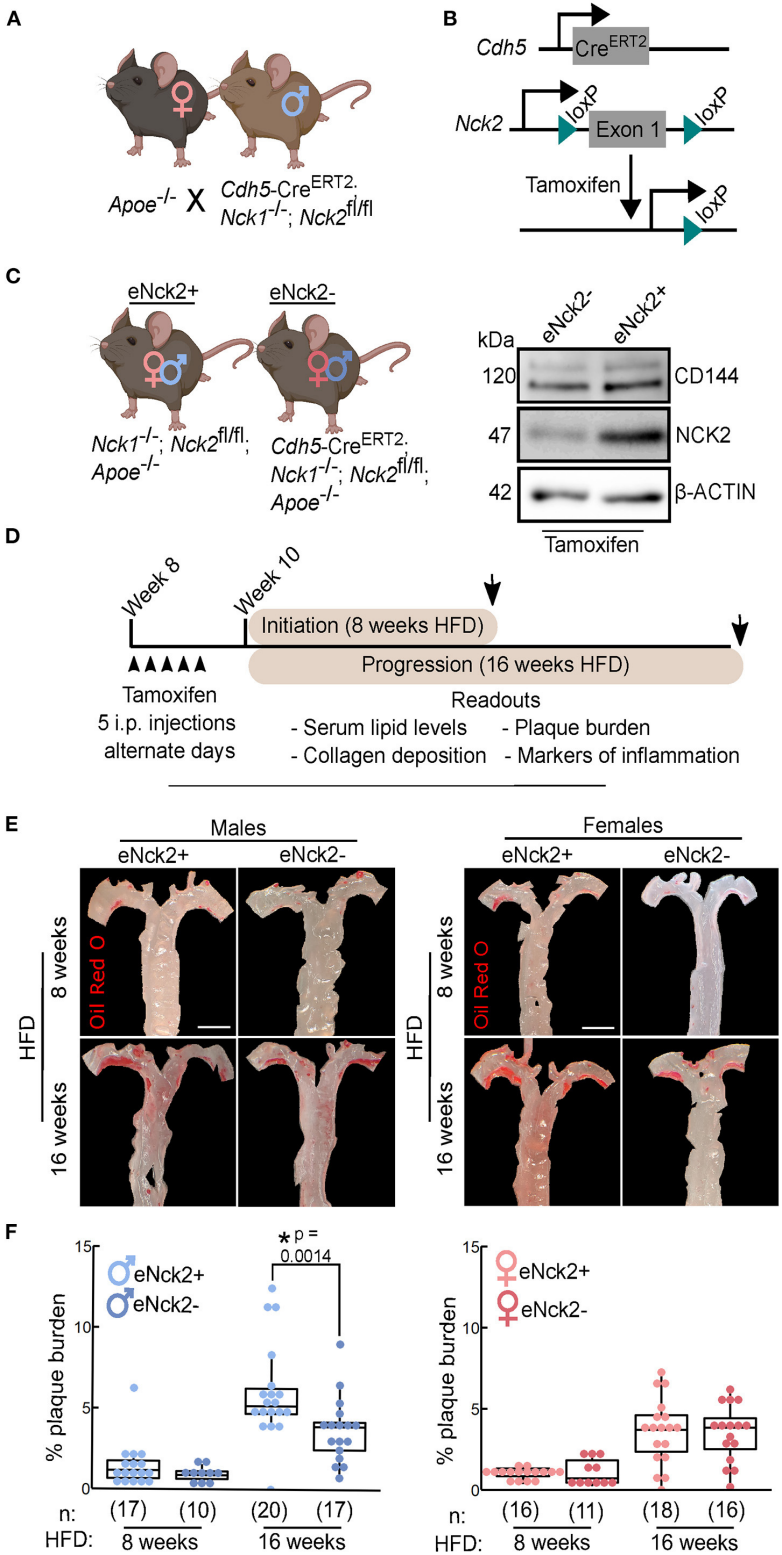
Assessment of atherosclerosis initiation and progression in atheroprone male and female mice with or without endothelial-specific deletion of NCK2. **(A)** APOE-deficient (*Apoe*^−/−^) mice were crossed with mice homozygous for both an *Nck1*-null allele and a floxed *Nck2* allele and that carried inducible Cre recombinase to selectively inactivate *Nck2* in the endothelium (*Cdh*5−*Cre*^ERT2^; *Nck*1^−/−^; *Nck*2^fl/fl^). **(B)** Tamoxifen-induced Cre recombination mediates excision of the loxP site-flanked exon 1 of *Nck2*. **(C)** Experimental groups consisted of male and female mice with (eNck2+) and without (eNck2-) NCK2 expression in the endothelium (left). Western blots using extracts from lung endothelial cells showing tamoxifen-induced deletion of NCK2 alongside levels of the endothelial marker CD144 (VE-cadherin) and the loading control β-ACTIN (right). **(D)** Diagram showing timeline (starting on week 8 after birth) of experimental manipulations, including tamoxifen injections (arrowheads), duration of high fat diet (HFD) regime, timing of tissue collection (arrows), and key readouts. **(E)** Representative images of *en face* preparations of mouse thoracic aortas stained with Oil Red O. Aortas were obtained from male (left) and female (right) mice fed HFD for 8 or 16 weeks to assess atherosclerosis initiation and progression, respectively. The scale bar represents 2 mm. **(F)** Box plots showing quantification of plaque burden as percentage of exposed intimal area occupied by lesions in male (left) and female (right) mice. Statistical significance was determined by two-way ANOVA followed by Tukey's multiple means comparison test. *p < 0.05. Numbers of animals/group (n) are indicated in parentheses.

**Figure 2:**
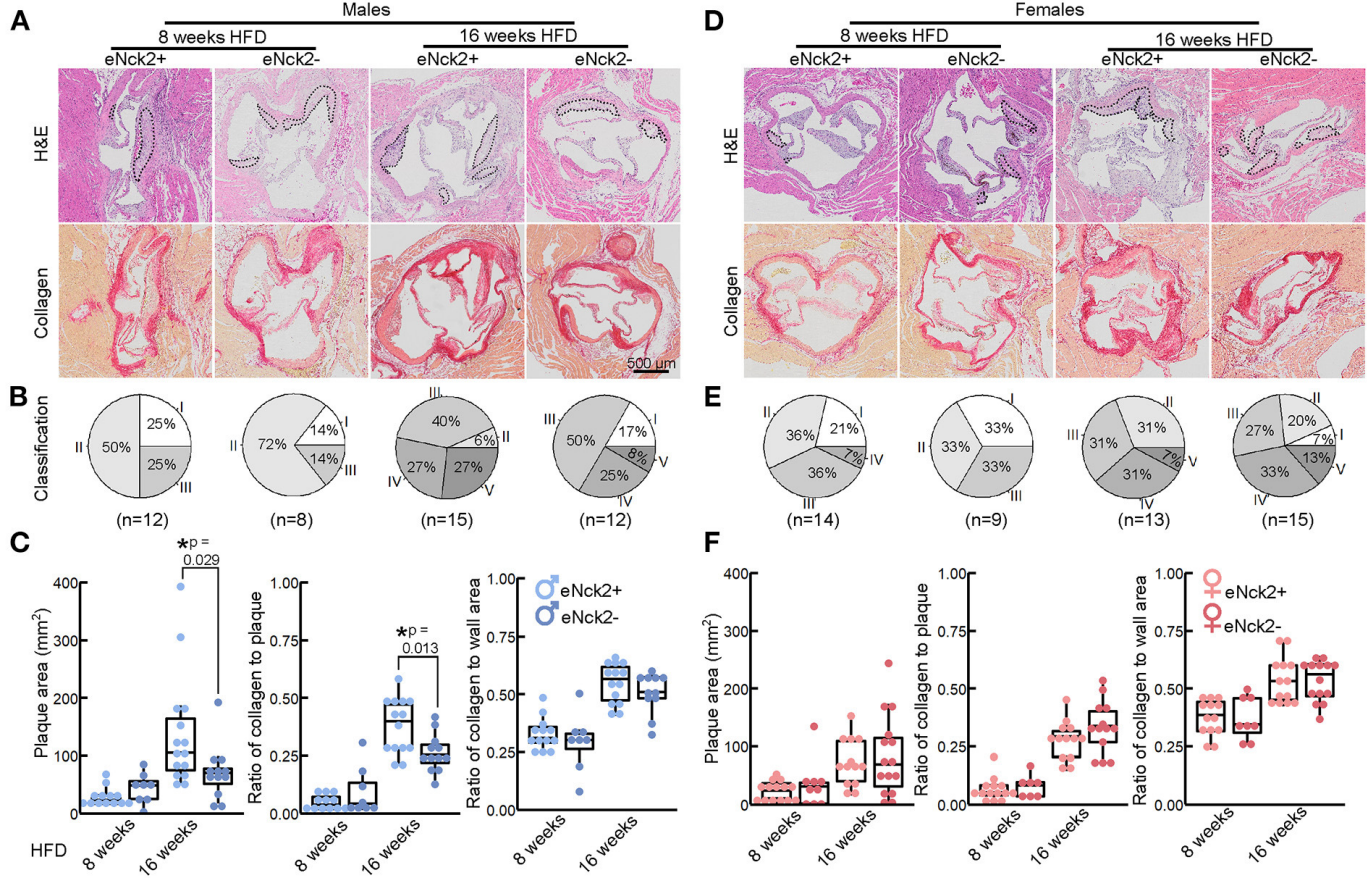
Morphometric analysis of aortic root lesions in atheroprone male and female mice with and without endothelial-specific deletion of NCK2. Animals were fed a HFD for 8 or 16 weeks to assess atherosclerotic lesion initiation and progression, respectively. **(A,D)** Representative images of aortic roots stained with H&E (top) and Picrosirius Red (bottom) to assess lesion development and collagen deposition, respectively. The scale bar represents 500 μm. **(B,E)** Pie charts showing percentage of lesion category in each experimental group. Lesions were classified as intima thickening (type I), fatty streaks (type II), intermediate lesion (type III), advanced atheroma (type IV), and fibroatheroma (type V). Numbers of animals/group are indicated in parentheses. **(C,F)** Box plots showing quantification of plaque area, ratio of collagen to plaque area, and ratio of collagen to aortic root wall area. Statistical significance was determined by Wilcoxon Rank Sum Test with Bonferroni correction. *p < 0.05.

We crossed APOE-deficient mice with mice homozygous for both an *Nck1*-null allele and a floxed *Nck2* allele, and that carried tamoxifen-inducible Cre recombinase expressed under control of the *Cdh5* promoter (Figure 1A). In these animals, tamoxifen-induced Cre activity results in *Nck2* inactivation by excision of the loxP site-flanked exon 1 (Figure 1B). The resulting progeny of mixed genetic background consisted of atheroprone mice either lacking (*Nck1*^−/−^*; Nck2*^*fl*/*fl*^*; Apoe*^−/−^) or carrying (*Cdh5*-Cre^ERT2^; *Nck1*^−/−^*; Nck2*^*fl*/*fl*^*; Apoe*^−/−^) Cre recombinase, herein termed eNck2+ and eNck2- (Figure 2C, left), respectively. Tamoxifen injection almost completely abolished NCK2 expression selectively in the endothelium of eNck2- but not eNck2+ animals (Figure 1C, right). To ascertain specific roles of NCK2 signaling and potential disease stage- and sex-dependent differences in endothelial inflammation and atherosclerosis, eight-week-old male and female mice from both genotypes were treated with tamoxifen and subsequently fed a HFD for 8 or 16 weeks to model atherosclerosis initiation and progression, respectively (Figure 1D). Serum triglyceride, HDL fraction, and total cholesterol levels did not differ by genotype or sex after 8 or 16 weeks of HFD (Supplementary Figure 1). Similarly, body weight gain did not differ by genotype or sex after 8 weeks of HFD. Regardless of genotype, however, males gained more weight than females after 16 weeks of HFD (Supplementary Figure 1). Plaque burden, determined in *en face* preparations of thoracic aortas stained with Oil Red O (Figure 1E), did not differ by genotype or sex after 8 weeks of HFD (Figure 1F). Although the overall plaque burden increased, only male mice lacking endothelial NCK2 (eNck2-) displayed decreased (*p* < 0.05) plaque burden compared to control eNck2+ animals after 16 weeks of HFD (Figure 1F). Thus, these results suggest an important, sex-dependent role for endothelial NCK2 in atherosclerosis progression but not initiation.

### Endothelial NCK2 increases plaque development and lesion severity in male mice

We used H&E and Picrosirius Red staining to analyze atherosclerotic lesion development and collagen deposition in aortic roots of male and female mice fed HFD for 8 or 16 weeks. Both lesion development and collagen deposition increased with prolonged exposure to HFD regardless of sex or genotype (Figures 2A,D). For each experimental condition, defined by the duration of HFD feeding, genotype, and sex, we determine the percentage of lesion type based on progression of pathological changes as previously described (19): intima thickening (type I), fatty streaks (type II), intermediate lesion (type III), advanced atheroma (type IV), fibroatheroma (type V). A slightly slower progression of pathological changes at initial disease stages (8 weeks of HFD) was seen in endothelial NCK2-deficient male (Figure 2B), but not female (Figure 2E), mice. As expected, advanced pathological changes were associated with the prolonged (16 weeks) regime of HFD. This pattern of lesion development was also seen in innominate arteries following 16 weeks HFD (Supplementary Figure 2). However, complete absence of early stage (type I) alongside a higher percentage of late stage (type V) lesions was observed in male (Figure 2B) but not female (Figure 2E) mice expressing endothelial NCK2 (male eNck2+ vs. eNck2-, *p* < 0.05). Furthermore, male eNck2+ mice developed significantly more plaque and accumulated more collagen in plaques than the eNck2- counterpart (Figure 2C, left & center panels). A larger proportion of eNck2+ vs. eNck2- males also developed lesions with clearly identifiable necrotic core after 16 weeks of HFD (Supplementary Figure 3). The incidence of other pathological findings, including media erosion and aortic dissection, did not differ by disease stage, sex, or genotype (Supplementary Figure 3). Although the ratio of collagen to wall area increased with the prolonged HFD regime in males, no genotype-dependent changes were observed (Figure 2C, right panel). In females, increase in plaque area, collagen content in plaques, and the ratio of collagen to wall area were dependent on the duration of the HFD regime but independent of genotype (Figure 2F). Consistent with the assessment of plaque burden (Figures 1E,F), analysis by histomorphometry shows that endothelial NCK2 promotes plaque development, i.e., increased plaque size and advancement of pathological changes, in male but not female atheroprone mice fed HFD for 16 weeks.

### Inactivation of endothelial NCK2 reduces expression of ICAM-1 in advanced disease stage

**Figure 3:**
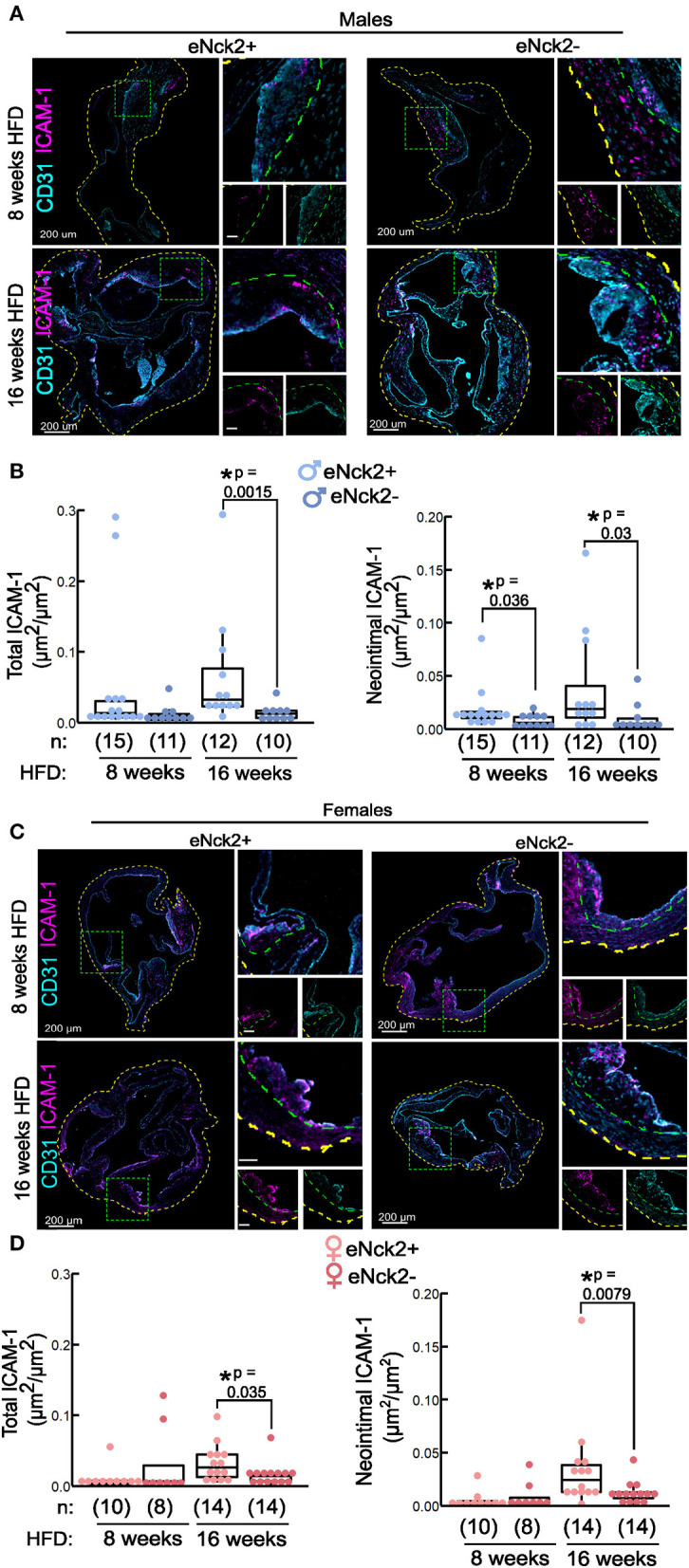
Determination of ICAM-1 in aortic roots of atheroprone mice with and without endothelial-specific deletion of NCK2. Animals were fed a HFD for 8 or 16 weeks to assess atherosclerotic lesion initiation and progression, respectively. **(A,C)** Representative confocal images of aortic roots showing immunofluorescence staining of endothelial (CD31) and cell adhesion (ICAM-1) markers in males **(A)** and females **(C)**. The dotted yellow, and green lines contour the aortic root section, and neointima layers, respectively. The scale bars represent 50 μm unless otherwise indicated. **(B,D)** Box plots showing quantification of total (left panels), and neointimal (right panels) ICAM-1 in males **(B)** and females **(D)**. Total ICAM-1 was calculated as ICAM-1 area in entire root/root area. Neointimal ICAM-1 was calculated as area of ICAM-1/neointimal area. Dots represent individual animals and the number of animals per group is indicated in parentheses. Statistical significance was determined by Wilcoxon Rank Sum Test with Bonferroni correction. *p < 0.05.

We performed co-staining of the endothelial and cell adhesion marker, CD31 and ICAM-1 respectively, in aortic roots obtained from male and female mice from both genotypes fed HFD for 8 or 16 weeks (Figures 3A,C). Total ICAM-1 expression, defined as the area of ICAM-1 signal in the entire aortic root normalized to the aortic root area, did not differ by genotype or sex at initial stages (8 weeks HFD) of disease (Figures 3B,D, left panels). In animals fed HFD for 16 weeks, however, inactivation of endothelial NCK2 decreased (eNck2+ vs. eNck2-, *p* < 0.05) total ICAM-1 expression in both males and females (Figures 3B,D, left panels). We assessed neointimal ICAM-1 expression, which was defined as the area of the ICAM-1 signal in the neointima normalized by the neointima area, which is delimited by the internal elastic lamina. At early stages of disease (8 weeks HFD), inactivation of endothelial NCK2 decreased neointimal ICAM-1 expression in males, but not females. At advanced stages of disease (16 weeks of HFD), inactivation of endothelial NCK2 decreased ICAM-1 neointimal expression in both males and females (eNck2+ vs. eNck2-, *p* < 0.05). In sum, endothelial NCK2 signaling promotes atherosclerosis progression, but not initiation, through a mechanism that involves increased expression of ICAM-1 in the arterial wall and neointimal/intimal layers.

### Endothelial Nck2 promotes vascular wall inflammation

**Figure 4:**
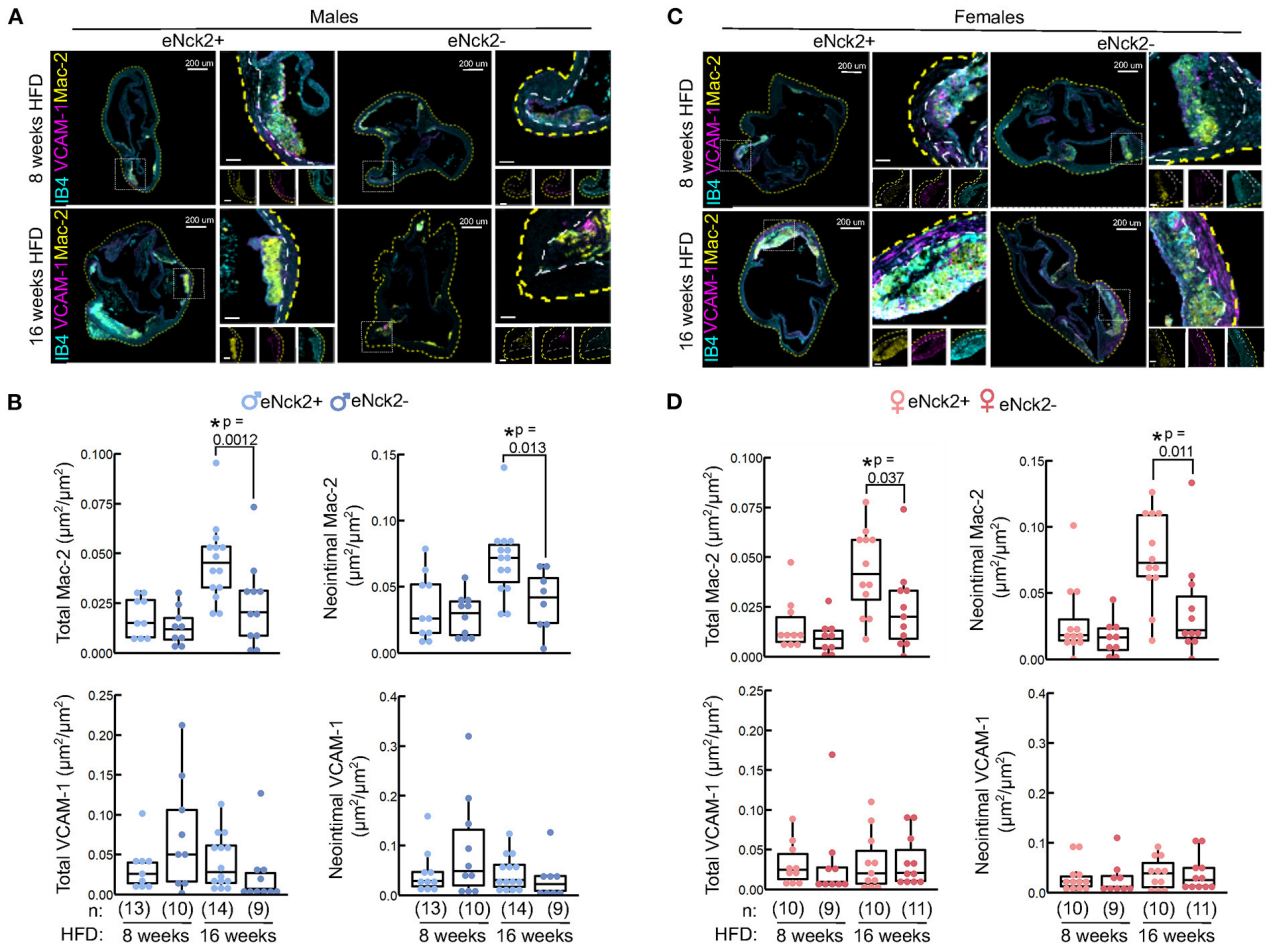
Inflammation in aortic roots obtained from atheroprone mice with or without endothelial-specific deletion of NCK2. Animals were fed a HFD for 8 or 16 weeks to assess atherosclerotic lesion initiation and progression, respectively. **(A,C)** Representative confocal images of aortic roots showing immunofluorescence staining of the cell adhesion (VCAM-1) and leukocyte infiltration (Mac-2) markers in males **(A)** and females **(C)**. The dotted yellow and white lines contour the aortic root section and the neointima, respectively. The scale bars represent 50 μm unless otherwise indicated. **(B,D)** Box plots displaying quantification of total Mac-2 or VCAM (left panels) and neointimal Mac-2 or VCAM-1 (right panels) in males **(B)** and females **(D)**. Total Mac-2 and VCAM-1 were calculated as Mac-2 or VCAM-1 area in entire root/root area. Neointimal Mac-2 and VCAM-1 were calculated as Mac-2 or VCAM-1 area in the neointima/area of neointima. Dots represent individual animals and the number of animals per group ins indicated in parentheses. Statistical significance was determined by Wilcoxon Rank Sum Test with Bonferroni correction. *p < 0.05.

To further assess inflammatory changes in the arterial wall, we co-stained VCAM-1 (inflammation marker), Mac-2 (macrophage infiltration), and the endothelium (IB4) in aortic roots obtained from male (Figure 4A) and female (Figure 4C) mice from both genotypes fed HFD for 8 or 16 weeks. We quantified total VCAM-1 expression (VCAM-1) and macrophage infiltration (Mac-2), respectively, as the area of VCAM-1 or Mac-2 signal in the entire root normalized to the root area. In addition, we quantified neointimal expression of VCAM-1 and macrophage infiltration as the area of VCAM-1 or Mac-2 signal in the neointima normalized to the neointimal area, which is delimited by the internal elastic lamina. Although enriched in atherosclerotic lesions, total (Figures 4B,D, bottom left panels) and neointimal (Figures 4B,D, bottom right panels) VCAM-1 expression did not differ by genotype, sex, or duration of HFD regime. Similarly, total and neointimal macrophage infiltration, assessed by Mac-2 staining, did not differ by genotype or sex in animals fed HFD for 8 weeks (Figures 4B,D). In contrast, in animals fed HFD for 16 weeks, total (Figures 4B,D, top left panels) and neointimal (Figures 4B,D, right panels) macrophage infiltration was significantly higher in both male and female control mice compared to those lacking endothelial NCK2 (eNck2+ vs. eNck2-, *p* < 0.05). Thus, endothelial NCK2 signaling exacerbates macrophage infiltration during advanced stages of atherosclerotic disease.

## Inactivation of endothelial NCK2 increases smooth muscle cell composition of plaques

To determine the effect of endothelial NCK2 on plaque stability, we co-stained αSMA (smooth muscle cell marker) and CD31 (endothelial marker) in aortic roots from male (Supplementary Figure 4A) and female (Supplementary Figure 4C) mice from both genotypes fed HFD for 8 or 16 weeks. We quantified smooth muscle cell infiltration as the area of αSMA signal normalized to plaque area. Plaque αSMA expression did not differ between genotypes (Supplementary Figures 4B,D) at 8 weeks; however, after 16 weeks HFD, plaques from eNck2− females trended (*p* = 0.074) towards a significant increase in αSMA signal in comparison to eNck2+ females. Taken together, endothelial NCK2 signaling promotes a plaque phenotype with increased smooth muscle cell contribution during atherosclerosis progression in females.

## Endothelial NCK2 has a differential effect on the atherogenic response of males and females

Presented data was further analyzed for sex-specific differences (Supplementary Figure 5). We observed a differential response between sexes in eNck2+ mice in (i) plaque burden of the thoracic aorta (Supplementary Figure 5A), (ii) aortic root plaque area (Supplementary Figure 5B, left), and (iii) collagen content of plaques in the aortic root (Supplementary Figure 5B, right) after 16 weeks HFD, as well as neointimal ICAM-1 levels after 8 weeks, but not 16 weeks HFD (Supplementary Figure 5C). No sex-specific differences were identified between male and female Nck2− mice in any of the tested responses at either initiation or progression. Analysis of the data in this fashion highlights the sex-specific effects of endothelial NCK2 in atherosclerosis.

## Discussion and conclusions

Targeting mechanisms underlying endothelial activation and dysfunction holds promise for the development of novel and more effective interventions for the prevention and treatment of atherosclerosis. Implementing a robust experimental design enabling analysis of atherosclerosis initiation and progression in both male and female *Nck1*-null atheroprone mice, we show here that endothelial NCK2 exerts disease stage- and sex-dependent effects on arterial inflammation and atherosclerosis development. Specifically in males, NCK2 signaling in the endothelium promotes HFD-stimulated vascular wall inflammation and atherosclerotic progression but not initiation. In females, however, these effects appear to be decoupled. Although also dispensable for atherosclerosis initiation, in advanced disease stages endothelial NCK2 signaling in females contributes to HFD-induced vascular wall inflammation but not plaque development. These results point to the increasingly recognized significance of sexual dimorphism and temporal regulation as critical factors underlying the complexities of atherosclerosis pathogenesis.

Sexual dimorphism in disease responses are increasingly appreciated within vascular pathologies (20, 21), including atherosclerosis (22). Our work specifically presents sex-specific differences in plaque area, plaque collagen composition, and neointimal ICAM-1 signal only in mice that retain endothelial NCK2 (Supplementary Figure 5). Surprisingly, although we observe several significant shifts in the male atherosclerotic phenotype with endothelial NCK2 deletion, the sole effect we observe in eNck2− females compared eNck2+ is a decrease in Mac-2+ area in the later stages of disease progression (Figure 4D). It is also worth noting that plaques in eNck2− females appear to develop with increased smooth muscle cell composition compared to eNck2+ females (Supplementary Figure 3B). Decreased macrophage content combined with increased smooth muscle cell infiltration suggests the establishment of a more stable plaque phenotype in female atheroprone Nck1-null mice with endothelial NCK2 deletion, a finding with potential clinical significance that merits further study. The highly similar NCK1 and NCK2 adaptors, sharing 68% amino acid identity, have overlapping functions during development (13). Expression of NCK2 in the endothelium compensates the global inactivation of *Nck1* during cardiovascular development (9) and postnatal angiogenesis (10). Our detailed analysis of *Nck1*-null atheroprone mice shows a significant role of endothelial NCK2 signaling in regulation of arterial wall inflammation, as evidenced by increased ICAM-1 expression and macrophage infiltration but not VCAM-1 expression. The absence of genotype-dependent differences in VCAM-1 expression in our study, is consistent with the highly variable expression of VCAM-1 in both intact areas and atherosclerotic lesions of the aortic root previously described in APOE-deficient mice fed HFD (23). Interestingly, despite being present in both sexes, decreased arterial wall inflammation translated into reduced plaque burden (en face aortas), plaque area and severity of atherosclerotic lesions (aortic roots) only in males lacking endothelial NCK2. Although the mechanism underlying the sex-specific differences in atherosclerosis progression described in this study remains to be elucidated, sexual dimorphism in NCK signaling appears to be an emerging theme (24).

Strengths of the present study include analysis of early and advanced stages of HFD-induced atherosclerosis and direct statistical comparison of readouts between sexes. Our results, however, appear at variance with those of recent studies examining the role of NCK signaling in atherosclerosis. Implementing the partial carotid ligation model of endothelial dysfunction and atherosclerosis (25), Orr and colleagues recently showed that endothelial NCK1, but not NCK2, mediates atheroprone flow-induced endothelial dysfunction/increased permeability (26). In a follow up study based on analysis of male mice, the same laboratory proposed a specific role for NCK1, but not NCK2, in atheroprone flow- and HFD-induced atherogenic inflammation and plaque formation (14). Results from the latter study are, however, difficult to interpret considering that (i) comparisons involved mice with different developmental levels of NCK, i.e., mice with constitutive, global inactivation of *Nck1* and mice with inducible, endothelial-specific inactivation of *Nck2* against controls expressing wild type levels of NCK1 and NCK2, (ii) a decreased inflammatory response was coupled to global *Nck1* inactivation; specifically, plasma levels of several proinflammatory molecules, including interleukin 1α, interleukin 1β, tumor necrosis factor α, and C-C motif chemokine 2 (MCP-1), were reduced in mice with constitutive, global NCK1 deficiency but not in control mice or mice with endothelial-specific abrogation of NCK2 signaling, and (iii) vascular inflammation and plaque burden were assessed at a single interval, 12 weeks of HFD, during disease progression We sought to establish a phenotype for plaque development in both males and females of the *Nck1*-null atheroprone mouse with or without inducible (post-developmental) endothelial-specific *Nck2* inactivation to allow for direct comparison. Thus, one limitation of the present study is that it does not explore the molecular underpinnings of the identified phenotype, which falls beyond of the scope of this brief research report. Full elucidation of the specific roles of NCK paralogues in vascular cell dysfunction and atherosclerosis pathogenesis warrants further investigation. To maximize impact, future studies should implement recommendations for the design and execution of preclinical studies of atherosclerosis to uncover mechanisms underlying initiation, propagation, and regression of atherosclerotic lesions (27), and enable well-powered, direct statistical comparison of sexes (22).

In conclusion, the present report identifies previously unappreciated roles of endothelial NCK2 signaling in stage- and sex-dependent modulation of atherosclerosis development.

## Data availability statement

The original contributions presented in the study are publicly available. This data can be found here: http://www.informatics.jax.org/allele/genoview/MGI:7316557.

## Ethics statement

The animal study was reviewed and approved by Texas A&M University Institutional Animal Care and Use Committee.

## Author contributions

GR conceptualized the study. BB maintained the mouse colony, implemented the experimental design, and performed necropsies and tissue collection. BB and GP processed tissues. BB, AT, and GR analyzed data and interpreted results. BB and GR prepared the manuscript. All authors approved the final version of the manuscript.

## Funding

This work was partly supported by AHA17GRNT33680067 and AHA19IPLOI34620007 grants (GR) and AHA829815 Predoctoral Fellowship (BB).

## Conflict of interest

The authors declare that the research was conducted in the absence of any commercial or financial relationships that could be construed as a potential conflict of interest.

## Publisher's note

All claims expressed in this article are solely those of the authors and do not necessarily represent those of their affiliated organizations, or those of the publisher, the editors and the reviewers. Any product that may be evaluated in this article, or claim that may be made by its manufacturer, is not guaranteed or endorsed by the publisher.

## References

[1] Libby P Buring JE Badimon L Hansson GK Deanfield J Bittencourt MS. Atherosclerosis. Nat Rev Dis Primers. (2019) 5:56. 10.1038/s41572-019-0106-z 31420554

[2] Dai G Kaazempur-Mofrad MR Natarajan S Zhang Y Vaughn S Blackman BR. Distinct endothelial phenotypes evoked by arterial waveforms derived from atherosclerosis-susceptible and -resistant regions of human vasculature. Proc Natl Acad Sci U S A. (2004) 101:14871–6. 10.1073/pnas.0406073101 15466704 PMC522013

[3] Gimbrone MA Jr Garcia-Cardena G. Endothelial cell dysfunction and the pathobiology of atherosclerosis. Circ Res. (2016) 118:620–36. 10.1161/CIRCRESAHA.115.306301 26892962 PMC4762052

[4] Ference BA Ginsberg HN Graham I Ray KK Packard CJ Bruckert E. Low-density lipoproteins cause atherosclerotic cardiovascular disease. 1 Evidence from genetic, epidemiologic, and clinical studies a consensus statement from the European atherosclerosis society consensus panel. Eur Heart J. (2017) 38:2459–72. 10.1093/eurheartj/ehx144 28444290 PMC5837225

[5] Libby P Everett BM. Novel antiatherosclerotic therapies. Arterioscler Thromb Vasc Biol. (2019) 39:538–45. 10.1161/ATVBAHA.118.310958 30816799 PMC6436984

[6] Bywaters BC Rivera GM. Nck adaptors at a glance. J Cell Sci. (2021) 134:18. 10.1242/jcs.258965 34558601 PMC10999758

[7] Chaki SP Barhoumi R Berginski ME Sreenivasappa H Trache A Gomez SM. Nck enables directional cell migration through the coordination of polarized membrane protrusion with adhesion dynamics. J Cell Sci. (2013) 126:1637–49. 10.1242/jcs.119610 23444376

[8] Chaki SP Barhoumi R Rivera GM. Actin remodeling by Nck regulates endothelial lumen formation. Mol Biol Cell. (2015) 26:3047–60. 10.1091/mbc.E15-06-0338 26157164 PMC4551318

[9] Clouthier DL Harris CN Harris RA Martin CE Puri MC Jones N. Requisite role for Nck adaptors in cardiovascular development, endothelial-to-mesenchymal transition, and directed cell migration. Mol Cell Biol. (2015) 35:1573–87. 10.1128/MCB.00072-15 25691664 PMC4387216

[10] Dubrac A Genet G Ola R Zhang F Pibouin-Fragner L Han J. Targeting NCK-mediated endothelial cell front-rear polarity inhibits neovascularization. Circulation. (2016) 133:409–21. 10.1161/CIRCULATIONAHA.115.017537 26659946 PMC4729599

[11] Chen J Leskov IL Yurdagul A Jr Thiel B Kevil CG Stokes KY. Recruitment of the adaptor protein Nck to PECAM-1 couples oxidative stress to canonical NF-kappaB signaling and inflammation. Sci Signal. (2015) 8:ra20. 10.1126/scisignal.2005648 25714462 PMC4413941

[12] Chen M She H Davis EM Spicer CM Kim L Ren R. Identification of Nck family genes, chromosomal localization, expression, and signaling specificity. J Biol Chem. (1998) 273:25171–8. 10.1074/jbc.273.39.25171 9737977

[13] Bladt F Aippersbach E Gelkop S Strasser GA Nash P Tafuri A. The murine Nck SH2/SH3 adaptors are important for the development of mesoderm-derived embryonic structures and for regulating the cellular actin network. Mol Cell Biol. (2003) 23:4586–97. 10.1128/MCB.23.13.4586-4597.2003 12808099 PMC164855

[14] Alfaidi M Acosta CH Wang D Traylor JG Orr AW. Selective role of Nck1 in atherogenic inflammation and plaque formation. J Clin Invest. (2020) 130:4331–47. 10.1172/JCI135552 32427580 PMC8011212

[15] Fawcett JP Georgiou J Ruston J Bladt F Sherman A Warner N. Nck adaptor proteins control the organization of neuronal circuits important for walking. Proc Natl Acad Sci U S A. (2007) 104:20973–8. 10.1073/pnas.0710316105 18093944 PMC2409251

[16] Jones N Blasutig IM Eremina V Ruston JM Bladt F Li H. Nck adaptor proteins link nephrin to the actin cytoskeleton of kidney podocytes. Nature. (2006) 440:818–23. 10.1038/nature04662 16525419

[17] Wang Y Nakayama M Pitulescu ME Schmidt TS Bochenek ML Sakakibara A. Ephrin-B2 controls VEGF-induced angiogenesis and lymphangiogenesis. Nature. (2010) 465:483–6. 10.1038/nature09002 20445537

[18] Venegas-Pino DE Banko N Khan MI Shi Y Werstuck GH. Quantitative analysis and characterization of atherosclerotic lesions in the murine aortic sinus. J Vis Exp. (2013) 82:e50933. 10.3791/50933 24335758 PMC4045007

[19] Vedder VL Aherrahrou Z Erdmann J. Dare to Compare. Development of atherosclerotic lesions in human, mouse, and zebrafish. Front Cardiovasc Med. (2020) 7:109. 10.3389/fcvm.2020.00109 32714944 PMC7344238

[20] Rudnicki M Abdifarkosh G Rezvan O Nwadozi E Roudier E Haas TL. Female mice have higher angiogenesis in perigonadal adipose tissue than males in response to high-fat diet. Front Physiol. (2018) 9:1452. 10.3389/fphys.2018.01452 30405427 PMC6206240

[21] Okuyama M Jiang W Javidan A Chen JZ Howatt DA Yang L. Lysyl oxidase inhibition ablates sexual dimorphism of abdominal aortic aneurysm formation in mice. Circulation. (2020) 142:1993–5. 10.1161/CIRCULATIONAHA.119.044986 33196308 PMC7678818

[22] Man JJ Beckman JA Jaffe IZ. Sex as a biological variable in atherosclerosis. Circ Res. (2020) 126:1297–319. 10.1161/CIRCRESAHA.120.315930 32324497 PMC7185045

[23] Zibara K Chignier E Covacho C Poston R Canard G Hardy P. Modulation of expression of endothelial intercellular adhesion molecule-1, platelet-endothelial cell adhesion molecule-1, and vascular cell adhesion molecule-1 in aortic arch lesions of apolipoprotein E-deficient compared with wild-type mice. Arterioscler Thromb Vasc Biol. (2000) 20:2288–96. 10.1161/01.ATV.20.10.2288 11031217

[24] Diab A Qi J Shahin I Milligan C Fawcett JP. NCK1 regulates amygdala activity to control context-dependent stress responses and anxiety in male mice. Neuroscience. (2020) 448:107–25. 10.1016/j.neuroscience.2020.09.026 32946951

[25] Nam D Ni CW Rezvan A Suo J Budzyn K Llanos A. Partial carotid ligation is a model of acutely induced disturbed flow, leading to rapid endothelial dysfunction and atherosclerosis. Am J Physiol Heart Circ Physiol. (2009) 297:H1535–43. 10.1152/ajpheart.00510.2009 19684185 PMC2770764

[26] Alfaidi M Bhattarai U Orr AW. Nck1, But not Nck2, mediates disturbed flow-induced p21-activated kinase activation and endothelial permeability. J Am Heart Assoc. (2020) 9:e016099. 10.1161/JAHA.120.016099 32468886 PMC7428973

[27] Daugherty A Tall AR Daemen M Falk E Fisher EA Garcia-Cardena G. Recommendation on design, execution, and reporting of animal atherosclerosis studies: a scientific statement from the American Heart Association. Circ Res. (2017) 121:e53–79. 10.1161/RES.0000000000000169 28729353

