# Supplementary figures and images for: Endothelial NCK2 promotes atherosclerosis progression in male but not female Nck1-null atheroprone mice
Source: Front Cardiovasc Med. 2022 Aug 12;9:955027. doi: 10.3389/fcvm.2022.955027 (PMC9413153; doi:10.3389/fcvm.2022.955027)

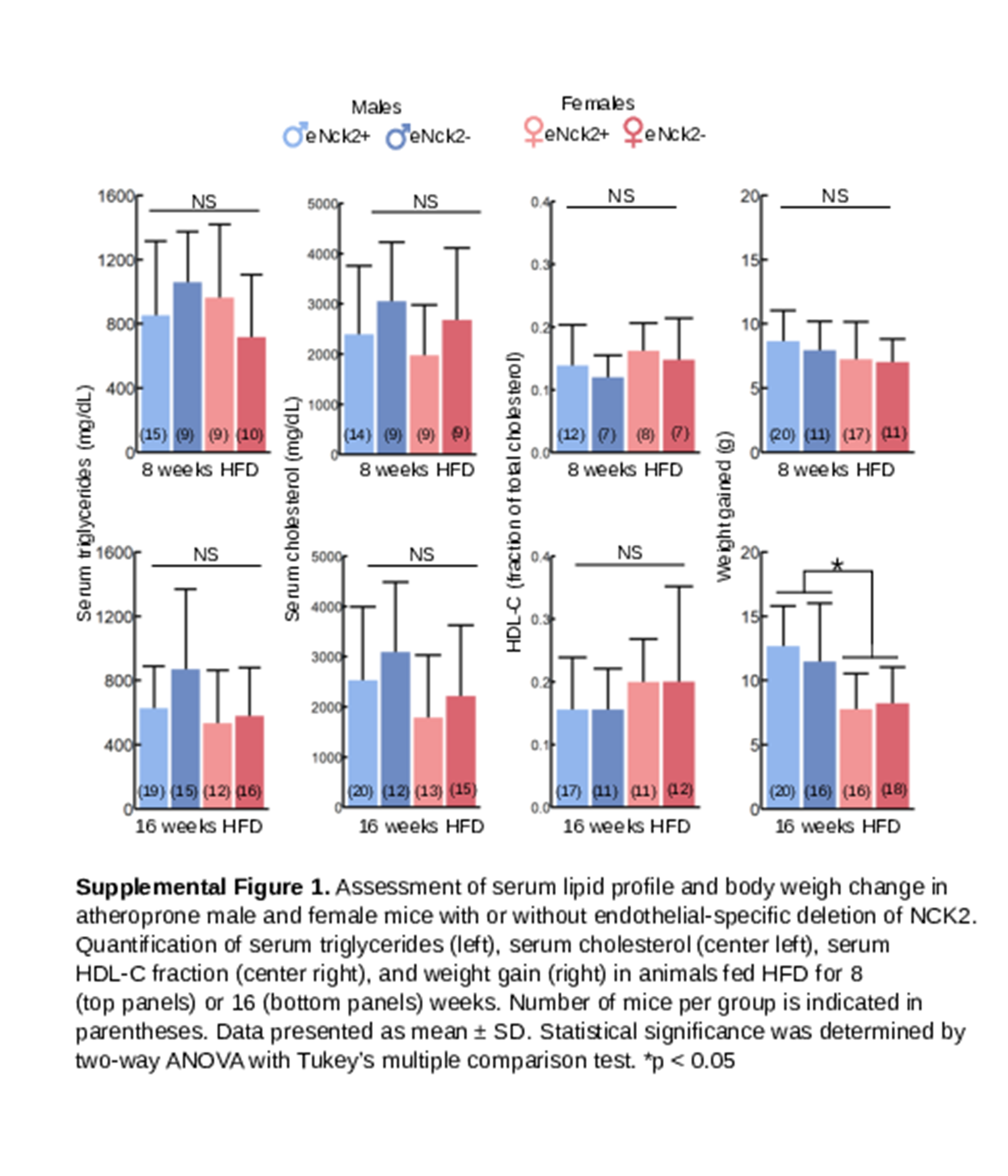

Supplement: Supplementary file 2 [file Image_1.tiff]

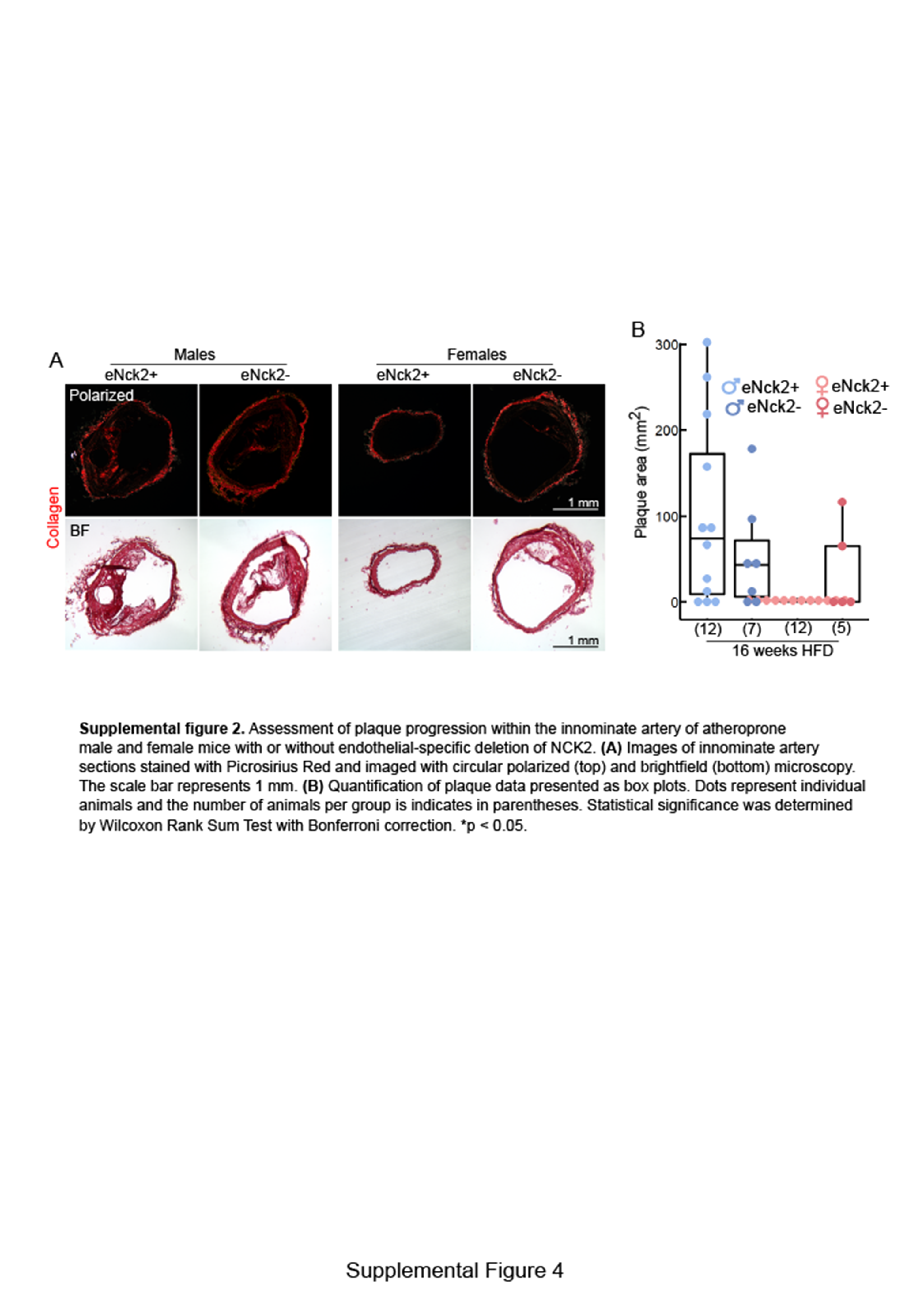

Supplement: Supplementary file 3 [file Image_2.tiff]

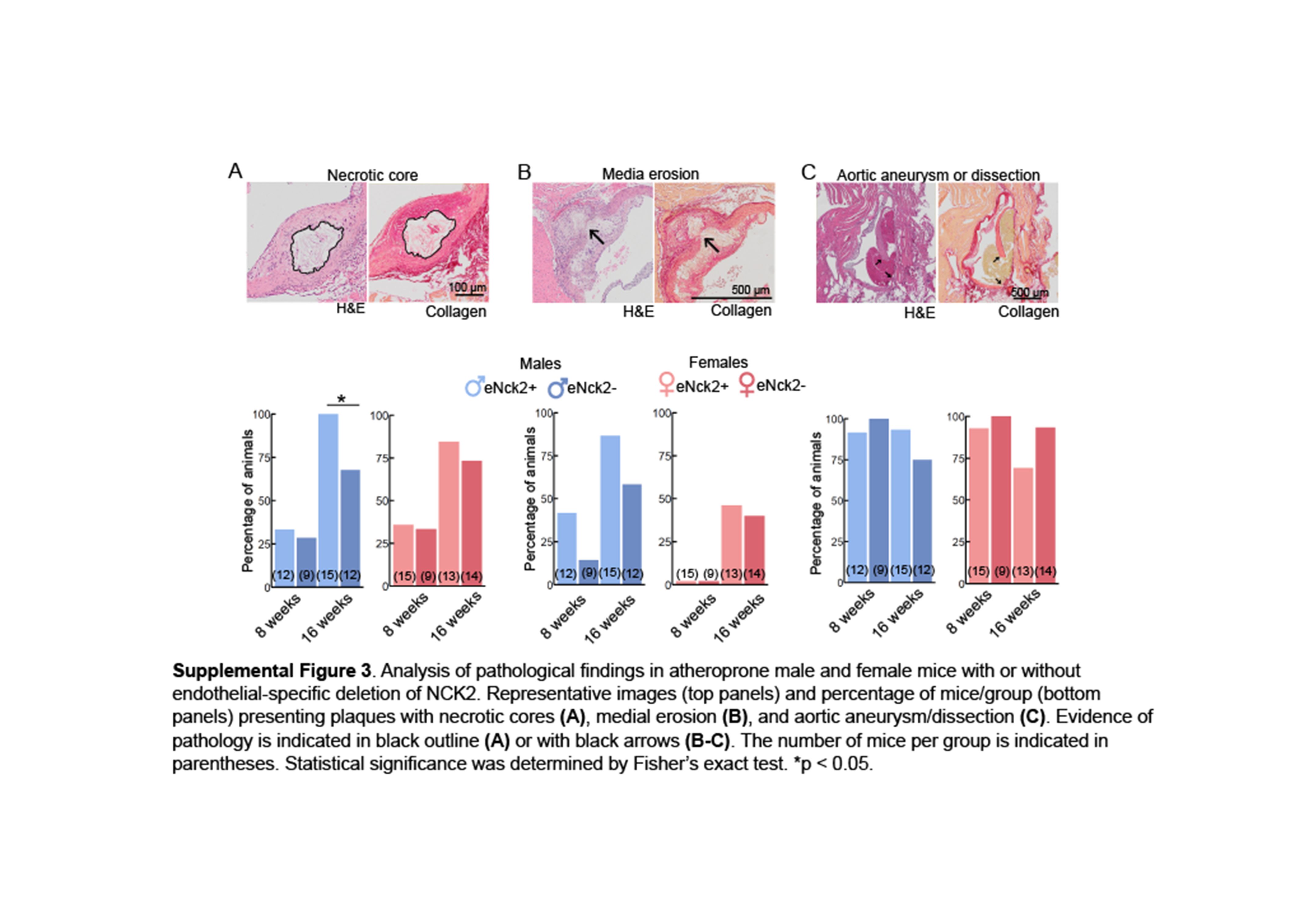

Supplement: Supplementary file 4 [file Image_3.tiff]

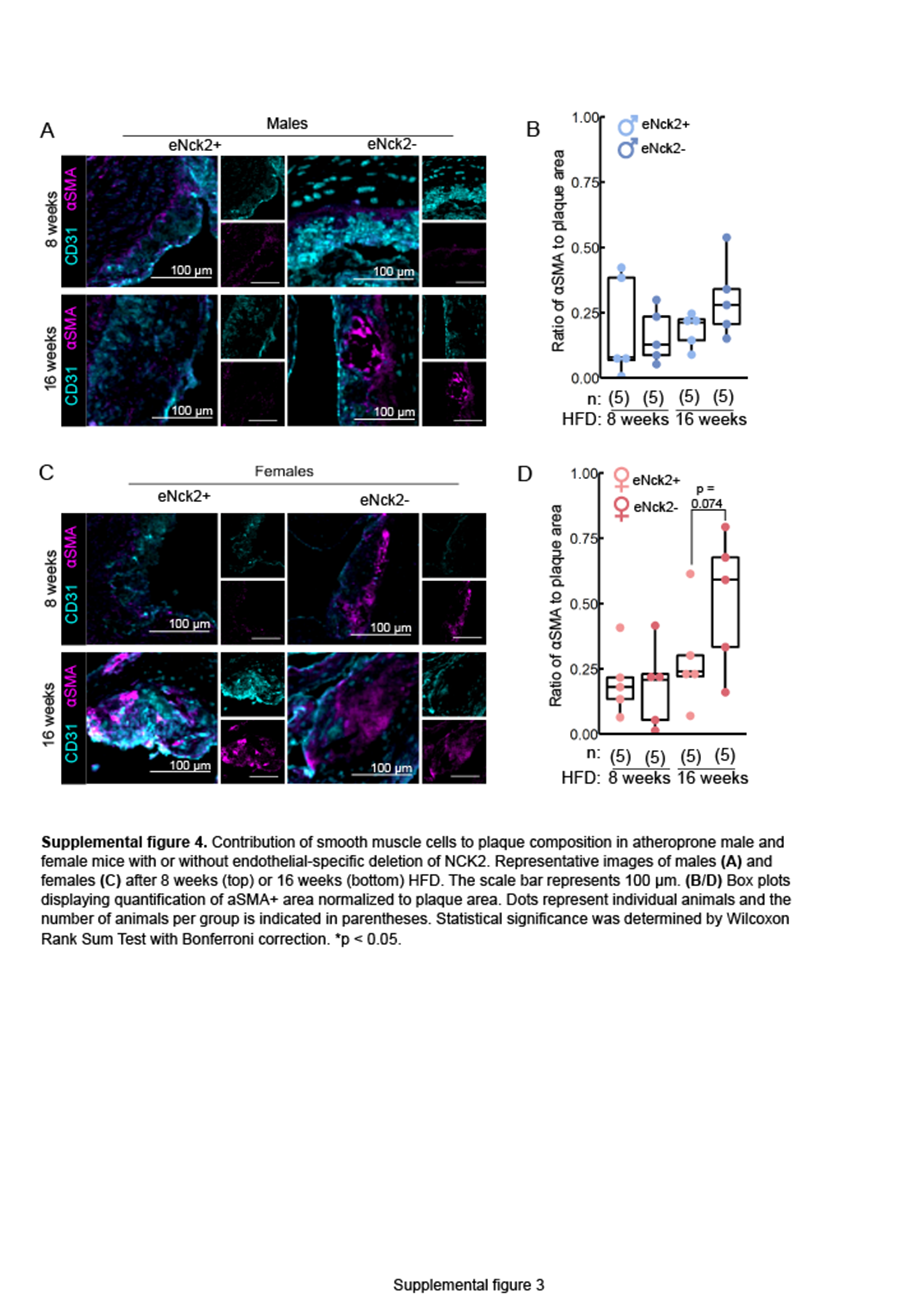

Supplement: Supplementary file 5 [file Image_4.tiff]

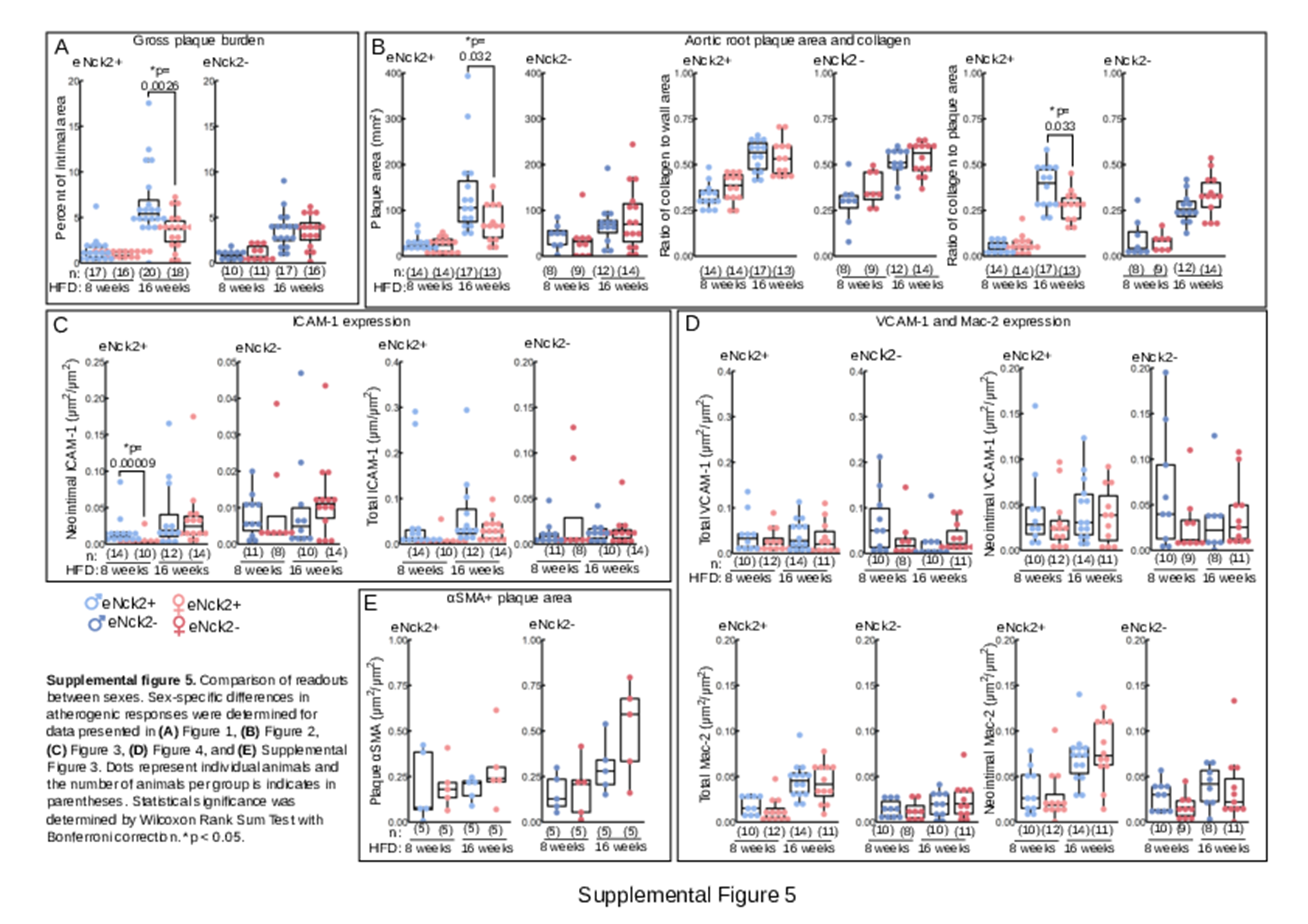

Supplement: Supplementary file 6 [file Image_5.tiff]
